# Ancient DNA Analysis Suggests Negligible Impact of the Wari Empire Expansion in Peru’s Central Coast during the Middle Horizon

**DOI:** 10.1371/journal.pone.0155508

**Published:** 2016-06-01

**Authors:** Guido Valverde, María Inés Barreto Romero, Isabel Flores Espinoza, Alan Cooper, Lars Fehren-Schmitz, Bastien Llamas, Wolfgang Haak

**Affiliations:** 1 Australian Centre for Ancient DNA, School of Biological Sciences, University of Adelaide, Adelaide, South Australia, Australia; 2 Proyecto Arqueológico Huaca Pucllana, Lima, Perú; 3 Department of Anthropology, University of California Santa Cruz, Santa Cruz, California, United States of America; 4 Max Planck Institute for the Science of Human History, Jena, Germany; Universita degli Studi di Pavia, ITALY

## Abstract

The analysis of ancient human DNA from South America allows the exploration of pre-Columbian population history through time and to directly test hypotheses about cultural and demographic evolution. The Middle Horizon (650–1100 AD) represents a major transitional period in the Central Andes, which is associated with the development and expansion of ancient Andean empires such as Wari and Tiwanaku. These empires facilitated a series of interregional interactions and socio-political changes, which likely played an important role in shaping the region’s demographic and cultural profiles. We analyzed individuals from three successive pre-Columbian cultures present at the Huaca Pucllana archaeological site in Lima, Peru: Lima (Early Intermediate Period, 500–700 AD), Wari (Middle Horizon, 800–1000 AD) and Ychsma (Late Intermediate Period, 1000–1450 AD). We sequenced 34 complete mitochondrial genomes to investigate the potential genetic impact of the Wari Empire in the Central Coast of Peru. The results indicate that genetic diversity shifted only slightly through time, ruling out a complete population discontinuity or replacement driven by the Wari imperialist hegemony, at least in the region around present-day Lima. However, we caution that the very subtle genetic contribution of Wari imperialism at the particular Huaca Pucllana archaeological site might not be representative for the entire Wari territory in the Peruvian Central Coast.

## Introduction

The Central Andes in South America are defined by marked geographic contrasts between the Andean highlands and the Pacific coast, with each of the regions being characterized by unique eco-geographic zones. The Central Andes are also closely associated with the history of iconic pre-Columbian South American civilizations such as the Wari, Tiwanaku and Inca [1–3]. The Wari and Tiwanaku represent culturally recognizable entities in the Central Andes during the Middle Horizon period (MH: 650–1100 AD), both with complex societies that are commonly associated with ecological changes, cultural transitions and accompanying population dynamics during this period [2, 3]. Archaeological records in the Central Andes have defined the MH as a dynamic time of demographic upheavals, involving interactions between highland and coastal populations, social stratification and socio-political changes [1, 4–8]. In particular, the Wari and Tiwanaku expansions triggered distinct cultural shifts across a vast area in the Central Andes during the Middle Horizon [1, 5, 6, 9, 10].

**Fig 1:**
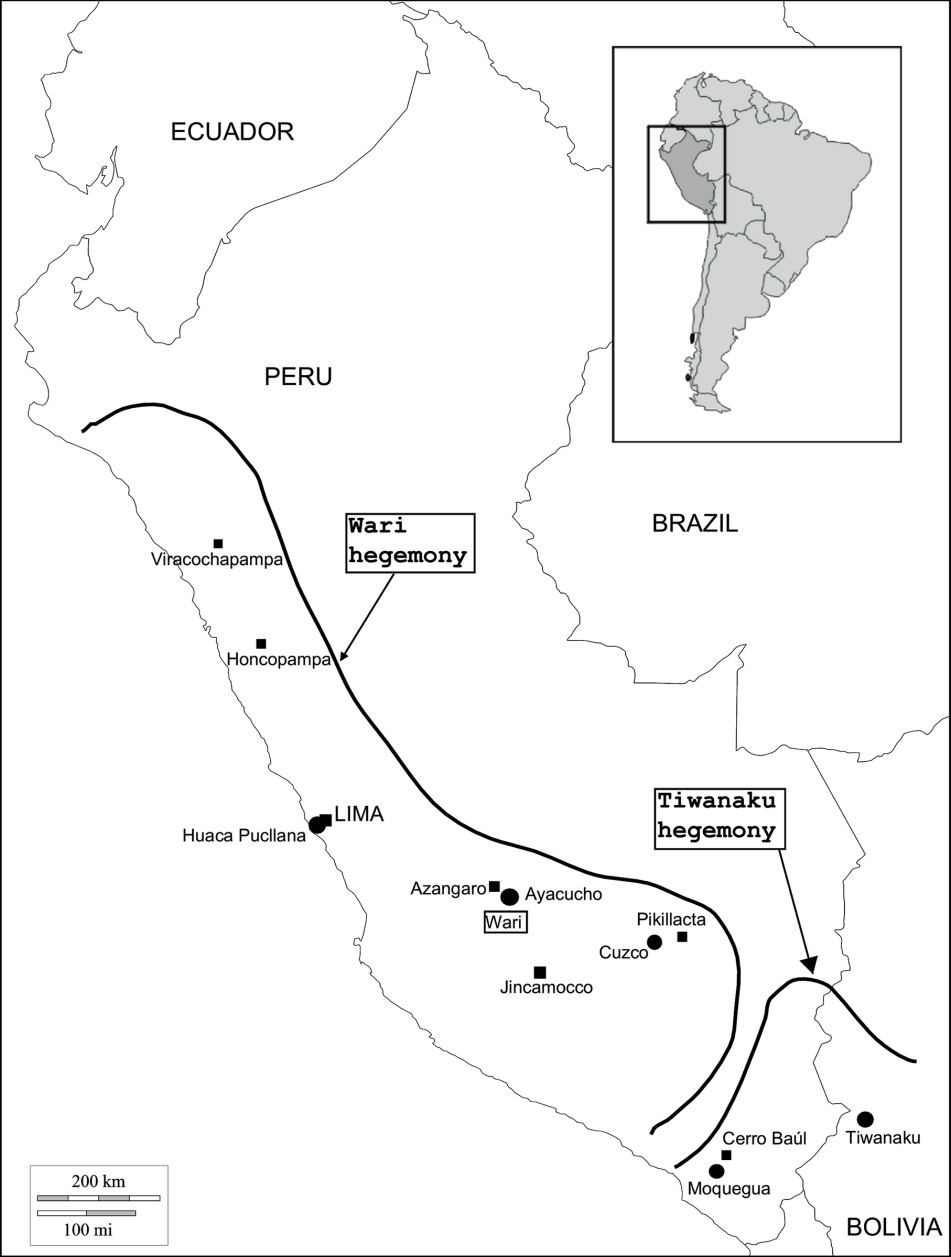
Map of Wari and Tiwanaku expansion in South America during the Middle Horizon. Adapted from [15].

The Wari Empire is considered the first imperial state in South America expanding from its political and urban center (i.e. Wari capital city) located in Ayacucho, Central highland Peru. The empire eventually covered a vast area of the Peruvian territory [5, 11, 12] holding a dominant position in administrative centers such as Viracochapampa, Willkawaín, Castillo de Huarmey in the Northern region of Peru, Honcopampa, Jincamocco Azangaro, Cerro Baúl in Moquegua, and Pikillacta in Cuzco [4, 8, 13, 14] (Fig 1).

Archaeological research has described the nature of the Wari Empire expansion [8] as a combination of religious indoctrination [16] and/or military campaigns [17] that ultimately led to the domination of other contemporary cultural groups such as Moche, Recuay and Nasca [8]. Scholars also argue that warfare and raids were political tools employed by the Wari to maintain imperial authority [18]. Populations were relocated to constitute workforces and develop “control cities” with defined architectural standards, a road network, and an extensive administrative system [4, 13, 17, 19]. However, there are aspects of Wari architecture and funerary practices in Peru’s Central Coast that are not found in other Wari settlements elsewhere in Peru [20]. Some scholars argue the Wari were a hegemonic imperialist state with presence in Peru’s Central Coast [6, 16, 21, 22], whereas others propose that Central Coast cities were commercial states that interacted with the Wari Empire, without being politically dominated or assimilated [23, 24].

Ancient DNA studies restricted to particular South American regions have clarified micro-evolutionary processes involved in the genetic composition of populations and their interaction with a particular environment [25–27]. However, the potential impact of the Wari imperialism has not been investigated genetically and it is unclear whether the political/cultural entity, i.e. “the Wari Empire”, also constitutes a biological entity, i.e. “the Wari people”. To investigate whether the Wari hegemony in the Peruvian Central Coast was based on expansion/replacement or on cultural diffusion, we generated a diachronic genetic dataset of complete mitochondrial genomes from three successive cultural periods at the Huaca Pucllana archaeological site in coastal Peru—following the site chronology: The Lima culture from the Early Intermediate Period—EIP (500–700 AD), the Wari culture from the Middle Horizon—MH (800–1000 AD), and the Ychsma culture from the Late Intermediate Period—LIP (1000–1450 AD). These three groups at Huaca Pucllana define a cultural transect in a restricted geographic area (present-day Lima), although other important settlements associated with the Wari expansion developed in the same region, such as Pachacamac, Cajamarquilla, Huaca San Marcos, Ancon, Huallamarca, and Catalina Huanca [21]. However, none of these archaeological sites display the cultural succession observed at Huaca Pucllana.

The aim of the study was to contrast the genetic diversity of the successive Lima, Wari and Ychsma cultures in a transect-through-time at the Huaca Pucllana archaeological site. We further analyzed the genetic makeup of Huaca Pucllana cultures in the broader context of South American past and present mtDNA diversity.

## Results

### Mitochondrial DNA haplogroups

From a total of 52 individuals collected from the Huaca Pucllana site, we obtained 34 complete mtDNA genome sequences (9 for Lima, 10 for Wari, 15 for Ychsma) using hybridization capture with RNA baits in solution and Illumina high-throughput sequencing. The average coverage depth per position was 97.4x (range: 16.8x-177.9x; Table 1). All 34 individual sequences encompass 28 distinct haplotypes, and were assigned to one of the main four Native American “founder” mtDNA haplogroups A2, B2, C1, and D4/D1 [28, 29]. A remarkable result of our study is that all ancient mtDNA sequences from Huaca Pucllana represent novel haplotypes not hitherto observed in modern-day populations, which harbor many additional variants from the common sub-haplogroups A2, B2, C1 and D4/D1 (Table 1).

**Table 1:** Haplogroup determination and mtDNA genomic coverage for Huaca Pucllana samples.

#	Sample number	Culture	Haplogroup	Coverage of the mitogenome	Average coverage depth per position (min–max)	StdDev	Additional mutations from the hg node	GenBank accession number
**1**	10791	Lima	A2	100%	122.7 (1–59)	13.5	C64T, 356.1C, T1189C, A5222G, T15289C, G16129A!, T16189C!	KU523266
**2**	10814	Lima	B2	100%	164.6 (9–186)	14.3	T2857C, T5082C, T5277C, A15924G, T16330C	KU523287
**3**	10802	Lima	B2	100%	63.4 (1–105)	14.4	C16168T	KU523286
**4**	10789	Lima	B2b	100%	100.2 (5–141)	15.1	G6261A, G9055A, A12972G, A14053G	KU523283
**5**	10817	Lima	C1b	100%	132.2 (5–169)	16.4	A9468G, C12535T, T15313C, A16166C, T16223C	KU523324
**6**	10820	Lima	C1b	100%	84.6 (1–135)	15.8	A9468G, C12535T, T15313C, A16166C, T16223C	KU523325
**7**	10806	Lima	C1c	100%	73.4 (1–117)	17.1	T152C!, 7472d, T8450C, C12774T, T16172C, T16297C	KU523322
**8**	10811	Lima	D4/ Pre-D1/D1	99. 99%	50.4 (0–97)	14.9	G143A, T2092C!, A6113G, T16189C!, G16274A, C16365T	KU523345
**9**	10821	Lima	B2	100%	98.2 (1–149)	19.9	T146C!,T3786C, A8170G, G15777A	KU523288
**10**	10774	Wari	A2	100%	67.7 (1–115)	18.1	C64T, A675G, A3354, T6911C, A13966G, C14800A, G16145A	KU523264
**11**	10754	Wari	B2b	100%	48.8 (1–99)	17	A243G, C4013T, G8994A, G11016A, G12127A, A14059G, G16438A	KU523282
**12**	10734	Wari	C1b	100%	59.8 (1–104)	17.3	C12535T, T15313C, A16166C, G16244T	KU523317
**13**	10763	Wari	C1b	99.90%	20.7 (0–55)	9	C12535T, T15313C	KU523318
**14**	10771	Wari	C1b	98.80%	18.2 (0–50)	8.2	A9468G, C12535T, T15313C, A16166C	KU523320
**15**	10773	Wari	C1b	100%	78.0 (0–119)	15.2	T8380C, T13281C, A13710C, A15244G, C15315T, A16207G	KU523321
**16**	10778	Wari	A2	97.60%	20.5 (0–100)	15.6	C64T, T1189C, T3786C, A5222G, C5765T, A7673G, T8738C, T15289C, C16221T, G12127A	KU523265
**17**	10765	Wari	C1b	99.80%	105.2 (0–340)	62.5	C12535T, T15313C, A16166C, G16244T	KU523319
**18**	10750	Wari	B2b	98.70%	22.3 (0–97)	16.7	T152C!, G1797A, A9377G, G12127A, A14053G, C14428T	KU523281
**19**	10742	Wari	B2b	100%	84.3 (0–248)	40.2	A178G, A6779G, A10420G, C16278T! C16295T	KU523280
**20**	10713	Ychsma	B2	100%	109.5 (2–163)	21.9	573.1C, A5186G, C5375T, C5895T, G15884A	KU523276
**21**	10729	Ychsma	B2	100%	101.6 (0–171)	24.8	T152C!, A2880G, C7786T, C13934T, A16051G, C16360T	KU523279
**22**	10720	Ychsma	B2b	100%	171.6 (19–191)	14.4	G6261A, G9055A, A14053G, C15647T	KU523277
**23**	10726	Ychsma	B2b	99.96%	38.5 (0–88)	14.7	G143A, T9078C, G10530A, A14053G, G16390A	KU523278
**24**	10709	Ychsma	C1b	100%	146.6 (0–176)	18.1	C12535T, T15313C	KU523313
**25**	10717	Ychsma	C1b	100%	175 (18–191)	11.9	C150T, T152C!, C12535T, T15313C, C16292T	KU523314
**26**	10725	Ychsma	C1b	100%	177.9 (0–192)	16.6	C12535T, T15313C	KU523315
**27**	10731	Ychsma	C1b	100%	182 (0–193)	17.7	A636G, C3442T, C4496T, G5821A, A5894C, G16000A, T16362C	KU523316
**28**	10722	Ychsma	D1	100%	140.8 (0–181)	21	T11365C, G11906A, T12481C, C12557T, A14665G	KU523342
**29**	10732	Ychsma	D4/ Pre-D1/D1	100%	146.6 (0–180)	18.1	G143A, T2092C!, A6113G, T16189C!, G16274A, C16365T	KU523343
**30**	10730	Ychsma	B2b	99.80%	46.0 (0–102)	20.2	T131C, C4013T, G13708A, T14634C, T15784C	KU523275
**31**	10805	Ychsma	D1	100%	159.5 (7–186)	13.1	T11365C, G11906A, T12481C, C12557T, A14665G	KU523344
**32**	10810	Ychsma	C1d	99.90%	90.3 (0–166)	39.4	A14122G	KU523323
**33**	10794	Ychsma	B2	99.60%	16.8 (0–54)	8.9	G8290A, A16066G	KU523284
**34**	10800	Ychsma	B2b	100%	127.6 (4–166)	17.2	T131C,C4013T, G13708A, T14634C, C14873A, T15784C	KU523285

Additional variants from the sub-haplogroup nodes compared to RSRS [30]. We generally follow the nomenclature of Phylotree.org [31], in describing variants: Reversions (back mutations) to an ancestral state are indicated with an exclamation mark (!), while 7472d and 356.1C denotes a deletion or insertion at the given nucleotide position, respectively.

**Fig 2:**
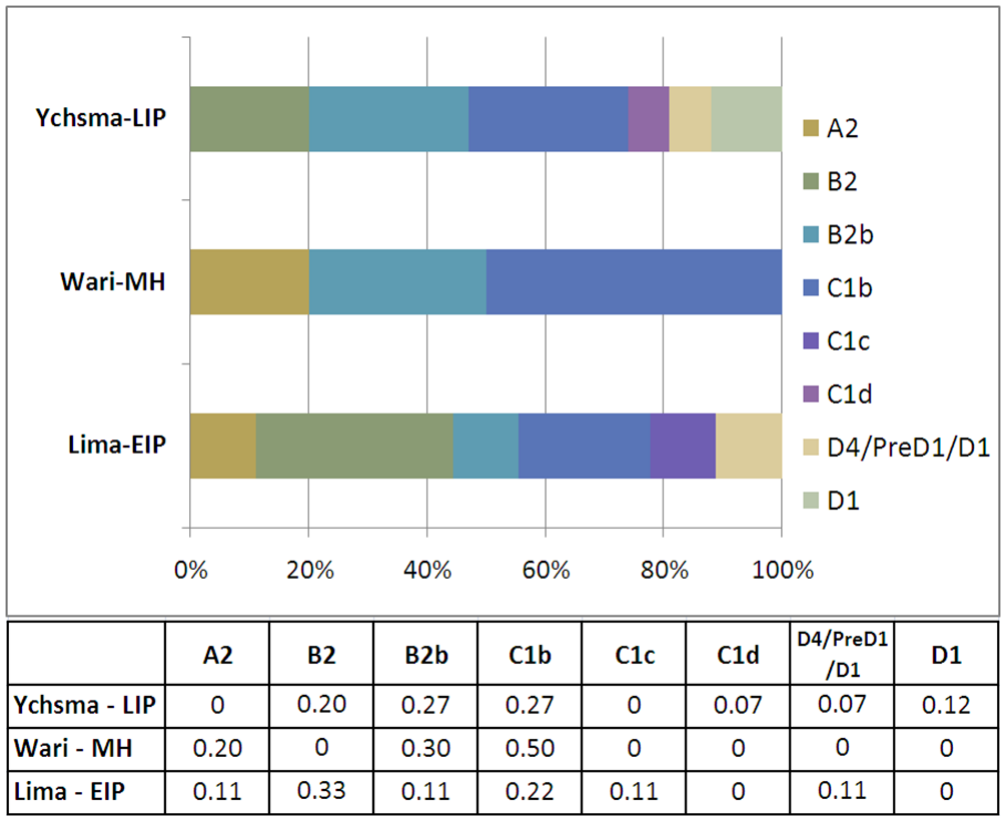
Mitochondrial DNA sub-haplogroup frequencies from Huaca Pucllana across cultural periods. Lima culture: EIP (500–700 AD), Wari culture: MH (800–1000 AD) and Ychsma culture: LIP (1000–1450 AD).

Detailed sub-haplogroup frequencies for the populations analyzed in this study were calculated per cultural period and are reported in Fig 2. We then used a Fisher exact test to investigate whether the sub-haplogroup composition varied significantly between the three cultural groups. However, all three comparisons returned non-significant p-values (Table 2).

**Table 2:** Summary statistics of ancient populations analyzed in this study.

	Population pairwise *F*_ST_ (upper diagonal) and p-values of the Fisher exact test (lower diagonal) *				Genetic diversity			Neutrality test			
	Lima	Wari	Ychsma	*N*	*H*	*h*	*π*	Tajima’s D	p-value	Fu’s FS	p-value
**Lima**	0	0.1905	0.6658	**9**	8	0.9722 (0.0640)	0.002331 (0.001272)	-0.13498	0.47000	1.44409	0.67400
**Wari**	-0.05197 (0.64865–1)	0	0.2144	**10**	9	0.9778 (0.0540)	0.002492 (0.001340)	0.18623	0.62400	1.08278	0.60900
**Ychsma**	-0.06111 (0.87387–1)	-0.00770 (0.32432–0.97296)	0	**15**	13	0.9810 (0.0308)	0.002159 (0.001117)	0.31343	0.66300	0.31237	0.51100

* p-values were adjusted for multiple comparisons (Bonferroni correction) using the R script p.adjust (The R Project for Statistical Computing, https://www.r-project.org/).

*N*: Number of individuals, *H*: Number of haplotypes, *h*: Haplotype diversity, *π*: Nucleotide diversity

### Summary statistics and haplotype sharing

Negative sequence-based genetic distances pairwise *F*_ST_ between the tree cultural strata Lima, Wari and Ychsma suggest genetically homogenous groups through time: -0.05197 for Lima–Wari, -0.06111 for Lima–Ychsma and -0.00770 for Wari–Ychsma (associated p-values were also non-significant after Bonferroni correction; Table 2). Haplotype diversity (h) also did not differ between the three cultures: Lima (0.9722), Wari (0.9778), and Ychsma (0.9810). Likewise, we did not observe substantial differences for nucleotide diversity (*π*): Lima (0.002331), Wari (0.002492) and Ychsma (0.002159). Genetic diversity indices and neutrality tests indicated that individuals of the Ychsma culture had a higher haplotype diversity 0.9810 (p>0.0308) than the two previous occupation phases. Values for Tajima’s D (neutrality test) only suggested a population expansion for individuals associated with the Lima culture (-0.13498), while for Fu’s FS values, neither of the three groups Lima (1.44409), Wari (1.08278) and Ychsma (0.31237) indicated a recent population expansion (Table 2).

**Fig 3:**
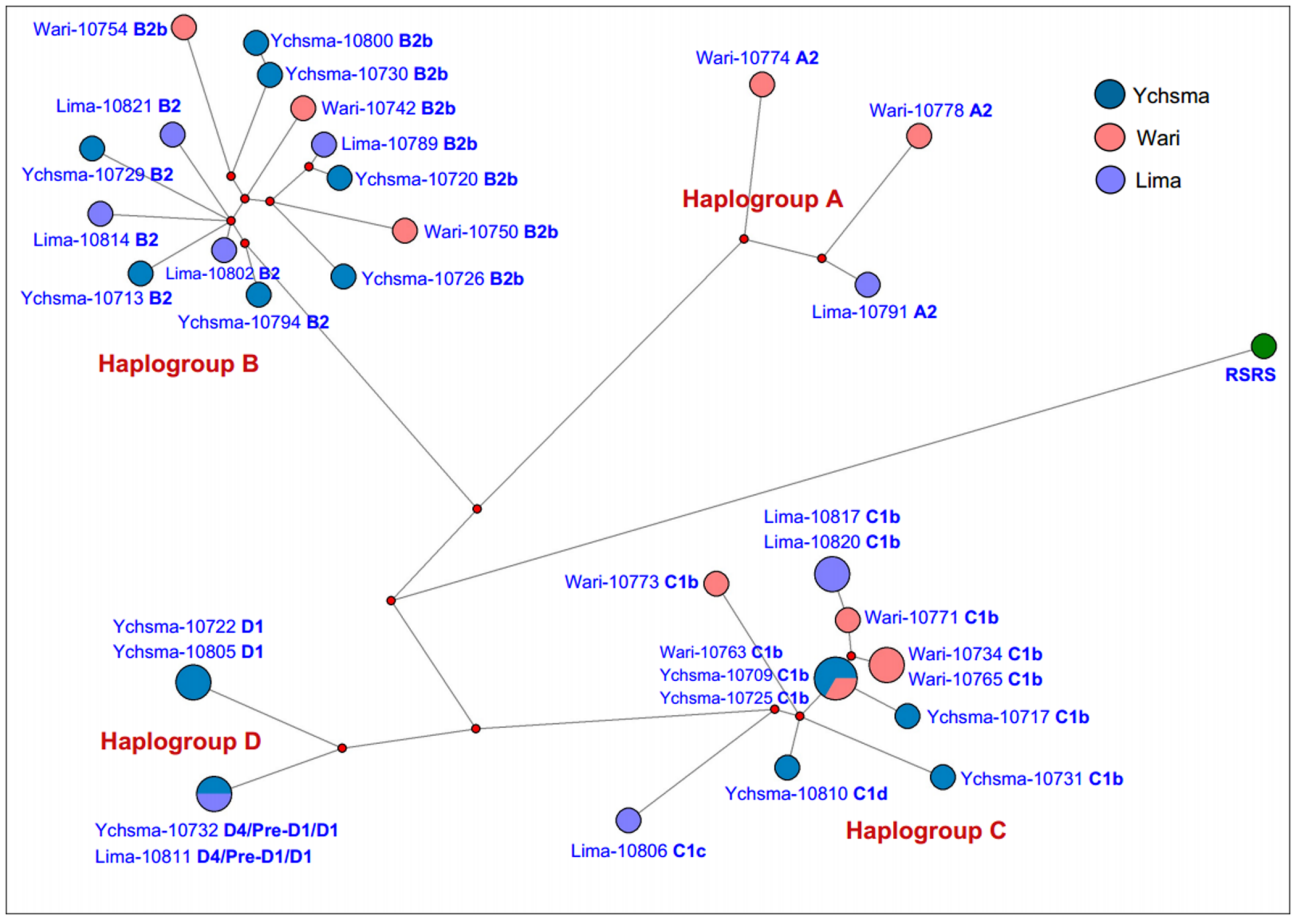
Phylogenetic network of mtDNA haplotypes from Huaca Pucllana.

In three instances, we found sequence haplotypes that were shared between individuals from the same cultural layer, such as two individuals from Lima (Lima–10817 / Lima–10820) belonging to mtDNA haplogroup C1b, two individuals from Ychsma (Ychsma–10722 / Ychsma–10805) belonging to D1, and two individuals from Wari (Wari–10734 / Wari–10765) belonging to C1b. We also observed shared haplotypes between individuals from different cultural layers, e.g. two individuals from Lima and Ychsma (Lima–10811 / Ychsma–10732) belonging to D4/Pre-D1/D1, and three individuals from Wari and Ychsma (Wari–10763 / Ychsma–10709 / Ychsma–10725) belonging to C1b (Fig 3).

### Huaca Pucllana mtDNA diversity in the South American context

We evaluated the relationship of cultural groups from Huaca Pucllana with modern and ancient mtDNA diversity in South America based on a genetic distance matrix and visualized as a Multidimensional Scaling Plot (MDS).

**Fig 4:**
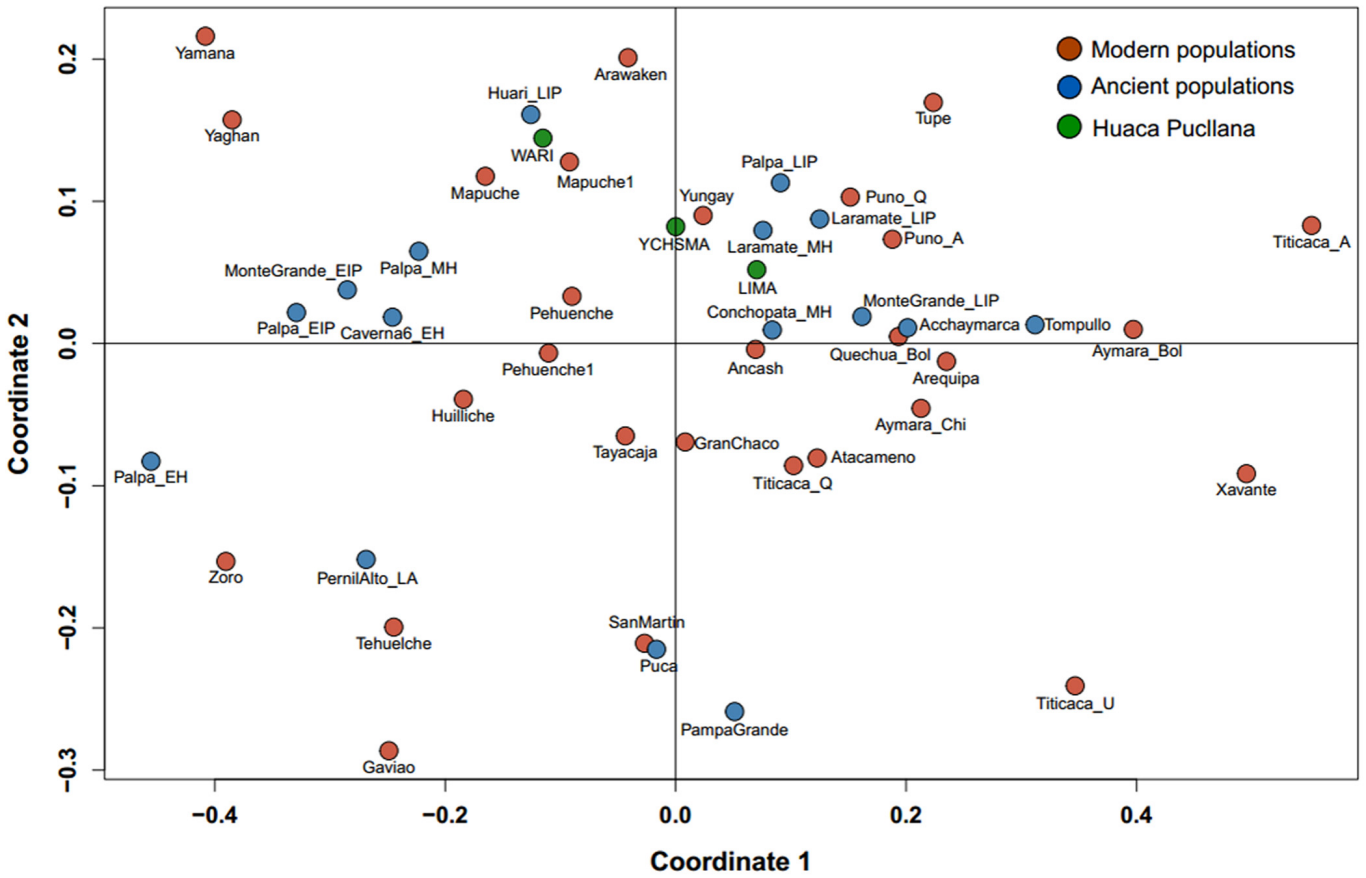
Multidimensional scaling plot (MDS) based on Slatkin’s *F*_ST_. Genetic affinities of Huaca Pucllana cultural groups compared with modern and ancient populations from South America.

Individuals from the EIP Lima culture are genetically close to highland Laramate populations from the MH [32] and LIP [33], and to Palpa coastal samples from the LIP [32]. Interestingly, ancient Lima differed completely from the southern coastal Nasca populations (Palpa EIP) from the same cultural horizon. Moreover, individuals from the MH Wari culture from Conchopata in the highlands also fall close to the coastal Lima individuals, whereas the Wari individuals from Huaca Pucllana have a strong affinity with Huari-LIP from the highlands [2] and two modern-day Mapuche groups (Mapuche and Mapuche1) from Chile [34, 35]. Individuals from the LIP Ychsma culture fall intermediate between the ancient Wari and Lima individuals and close to modern-day Yungay populations from Peru [36] (Fig 4).

## Discussion

We sequenced 34 complete human mitochondrial genomes from the three successive cultural periods Lima, Wari and Ychsma found at Huaca Pucllana. This dataset represents a unique chronological transect in the Peruvian Central Coast.

Low and non-significant *F*_ST_ values for comparisons between the three groups Lima, Wari and Ychsma indicate no differentiation of sequence haplotypes, meaning that ancient Peruvians from Huaca Pucllana did not undergo dramatic changes in genetic ancestry over approximately ~1000 years. These results suggest population continuity through time, with no major demographic turnovers. Genetic diversity indices do not differ substantially between the three cultural periods and results from neutrality tests (Tajima’s D and Fu’s FS) do not attest for recent expansion in any of the three groups. Overall, samples from the latest Ychsma period are the most diverse, which could be explained by the slightly larger sample size (Table 2).

However, mitochondrial sub-haplogroup frequencies reveal subtle shifts between the three cultural periods. We observed a loss of basal haplogroup B2 lineages during the transition from Lima to Wari, alongside a slight increase of B2b lineages, suggesting the arrival of the latter in the Huaca Pucllana region during the occupation phase of the Wari Empire. Interestingly, the fact that basal B2 lineages (non-B2b) are present in subsequent LIP Ychsma individuals, while being absent in MH Wari, suggests a potentially exogenous source of the Wari B2b lineages (Fig 2).

In addition, half of the MH Wari individuals carried the C1b sub-haplogroup, which is twice as much as the earlier EIP Lima or later LIP Ychsma groups. Since the frequency of haplogroup B2b remains constant during the Wari and Ychsma periods, we suggest that the increase in frequency of haplogroups C1b and B2b during the Middle Horizon might be linked to gene flow from the Andean region. However, we note that haplogroup A2 lineages also increase with the Wari period, but do not seem to become established in the Huaca Pucllana region during Ychsma times, following the collapse of the Wari Empire (Fig 2).

We caution that haplogroup composition represents a very broad approach to investigate signals of population structure, in particular because frequencies are subject to genetic drift or stochastic processes, population movements and gene flow [37]. Moreover, sample assignation was confirmed by radiocarbon dates for only a subset of specimens from the three cultural periods. It is therefore possible that some of the undated specimens may have been misassigned to a particular cultural layer and results may be interpreted with care.

Network analysis showed that haplotype sharing occurred mostly between individuals from the same archaeological period, and less often between different cultural layers (only two instances, i.e. Wari–Ychsma, Lima–Ychsma). A shared C1b lineage between Wari and the subsequent Ychsma is very plausible, while the shared Lima–Ychsma haplotypes suggests a local continuity of particular maternal lineages over several centuries, which implies that the Wari incursion was not a replacement but rather brought new lineages to the Huaca Pucllana region (Fig 3).

Interestingly, the MDS plot shows an affinity of the Lima population during the EIP (i.e. before the arrival of Wari) in Peru’s Central Coastal with ancient highland populations rather than other Coastal populations from the same period (i.e. Palpa EIP and Montegrande EIP), suggesting that the population history of the Central Coast is connected with the highlands and did not develop independently unlike Moche and Nasca populations [38]. However, this observation is independent from the archaeological record of the Central Coast and should not be over-interpreted in terms of cultural origins of the Lima Culture. The clustering of the Wari individuals from Huaca Pucllana with Huari-LIP from the highlands suggests a genetic similarity of the Wari group from the MH with the post-Wari LIP individuals in the Central Andean region in Peru. The persistence of Wari lineages in individuals from the subsequent Ychsma culture explains the intermediate position of the Wari between the Ychsma and the Lima substratum and could be considered as legacy of the Wari influence (Fig 4).

It should be noted that the overall mtDNA haplogroup composition is nonetheless homogenous across cultural periods in Peru Central Coast, even in the light of the expansion of the Wari Empire. To the limits of our resolution, the new results from ancient Huaca Pucllana therefore hint at a very subtle yet negligible genetic contribution of highlands Wari in coastal Peru during the MH.

We caution that the situation at Huaca Pucllana in the greater Lima region might not be representative of the overall genetic influx of the Wari Empire in the Central Coast of Peru, supporting the hypothesis that local settlements along the Central Coast were not entirely dominated or affected by Wari imperialism [23, 24]. Moreover, it appears that the Wari did not adopt highly aggressive means of control such as direct colonization outside the political center in Ayacucho, as suggested previously [39]. In this context, we suggest that the independence of local and regional identities during the MH must have remained intact, and thus implies group interactions and a level of political and social complexity which has not yet been fully explored in the Central Coast of Peru [39]. Consequently, definitive assertions about the Wari imperialism/colonialism in Peru’s Central Coast require further research.

A remarkable observation is that the ancient mtDNA sequences from Huaca Pucllana represent exclusively novel haplotypes (Table 1) with many variants additional to the common sub-haplogroups A2, B2, C1 and D4/D1. This suggests that the pre-Columbian genetic diversity was previously underestimated, which can be explained by two complementary premises: i) the mtDNA diversity of pre-Columbian populations was substantially higher than in modern-day Native populations; and ii) there was a considerable loss of pre-Columbian mtDNA diversity as a result of the dramatic demise of Native Americans during and after the European colonization [40]. Alternatively, it is also possible that the modern genetic diversity is largely underestimated, and that sequencing mitochondrial genomes from relevant Central Andean populations might reveal direct matches with samples from colonial times, and today.

As exemplified by the pre-Columbian expansions of Wari and Tiwanaku and later on the Inca Empire, the Central Andes have always been a dynamic environment with considerable population movements [1]. Another large-scale internal migration movement took place in Peru in more recent times, with rural and indigenous population (e.g. Junín, Ayacucho, La Libertad, Ica, Lambayeque, Cajamarca, Piura, and to a lesser degree from other places) moving towards the urban centers like Lima since the 1940's [41]. It is therefore likely that the ancestry of people from large urban centers like today’s Lima must have been constantly receiving additional genetic diversity.

## Conclusion

The ~1000-year transect through time of pre-Columbian history at Huaca Pucllana, allowed us to test the impact of the expansion of the highland Wari Empire on the coastal Lima culture, and its legacy in the subsequent Ychsma culture. The results from three archaeological periods indicate that genetic diversity in Huaca Pucllana shifted only slightly through time, ruling out a complete population discontinuity or replacement driven by the Wari imperialist hegemony, at least in the region around present-day Lima.

Detailed chronological sampling and high-resolution DNA sequencing efforts are required to refine the analysis of ancient genetic diversity in Peru, but importantly also from modern-day groups from Central Andean and Coastal regions to be able to contrast ancient diversity with present-day populations. Such data would allow the complete contextualization of associated meta-data, testing of demographic scenarios, and exploration of continuity/discontinuity, replacements and bottlenecks on the basis of statistical inferences or simulations. Ultimately, these analyses could elucidate the dynamic of pre-Columbian societies and the dramatic demise of Native American people that coincided with the time of the European arrival [42, 43].

## Materials and Methods

Ethics Statement: All necessary permits were obtained for the described study, which complied with all relevant regulations. Permissions to collect, export and analyze ancient Peruvian specimens from the Huaca Pucllana site were granted by the Ministry of Culture (the former National Institute of Cultural Heritage–INC) and the National Museum of Archaeology, Anthropology and History of Peru (MNAAHP) and are available on request (ACTA No 017-2010-ARMC-MNAAHP-MC and Resolución Viceministerial No. 120-2010-VMPCIC-MC).

### Huaca Pucllana archaeological context

**Fig 5:**
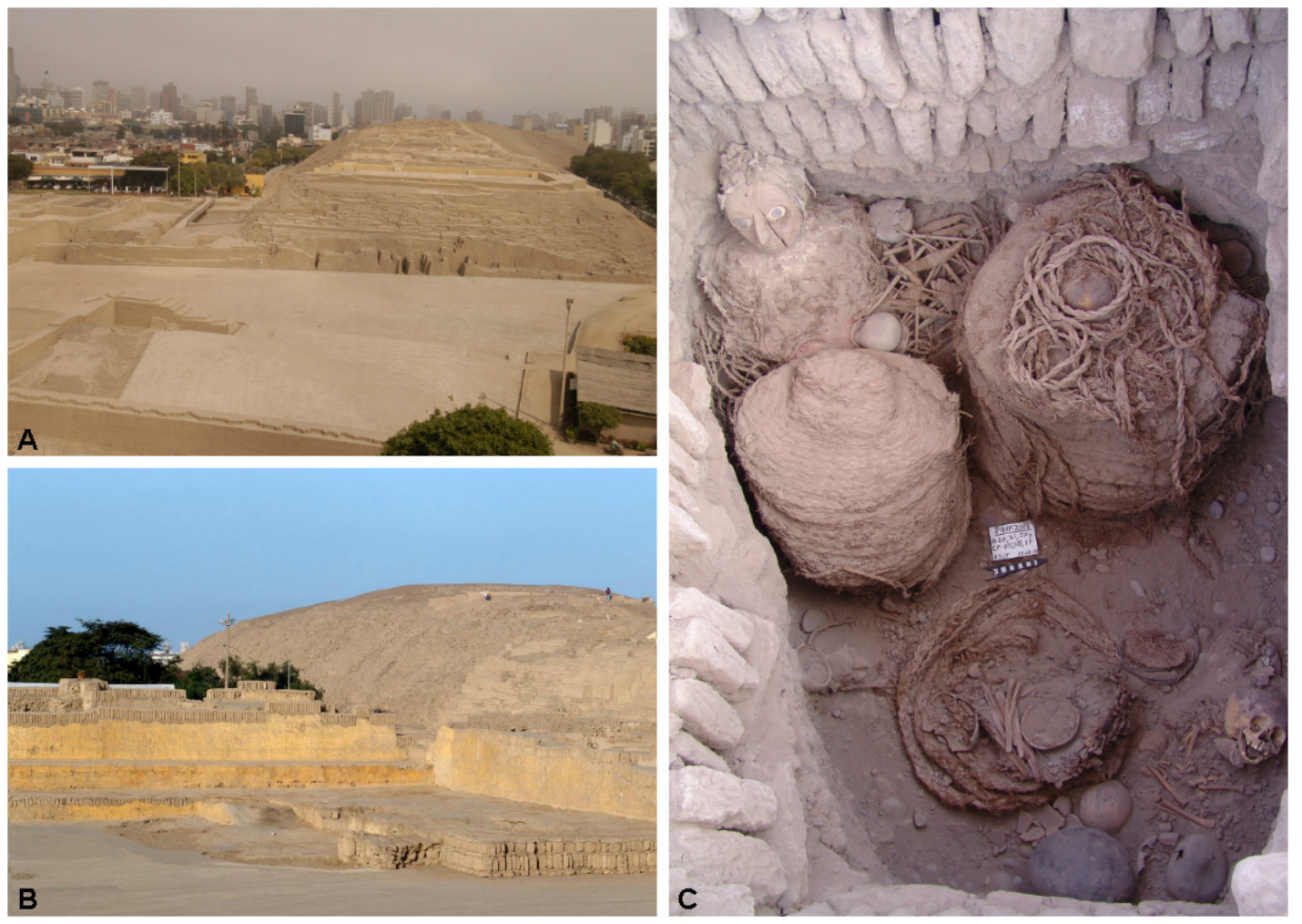
(A-B) View of the Huaca Pucllana archaeological site in Lima, Peru. (C) Wari funerary fardo “La Dama de la Máscara” (Credits: Huaca Pucllana research. Conservation and revalorization project).

The archaeological site of Huaca Pucllana (12°06’37.01”S–77°01’58.93”W) is located in the Miraflores District of Lima, Peru (Fig 5).

Archaeological evidence at the site defined that three cultural layers developed during slightly different times than in the classical chronology for the Central Andes established by Rowe [44]. The Lima culture (500–700 AD) occupied the site during the late EIP and early MH. The Wari culture established as a state in Ayacucho ~600 AD but reached the Central Coast and developed at Huaca Pucllana only later, around 800–1000 AD [45]. Finally, the time of the Ychsma culture (1000–1450 AD) is concordant with the classical chronology [46].

The construction of the Huaca Pucllana ceremonial center was done between the 4^th^ and 7^th^ centuries AD, which represents the Lima final phase (500–700 AD) and occupation of the site. Huaca Pucllana is characterized by pyramidal platforms built with sun-dried bricks, or adobes [46, 47]. The main structure at the site is the Great Pyramid, which is 400 m long, 60 m wide, 20 m high, and includes seven platforms [48–50]. It was used for various activities related to the worship of divinities associated with the so-called Sea Culture, with constant pilgrimage of people from surrounding regions. Human remains excavated in Huaca Pucllana were often associated with cultural elements that could be attributed to a particular layer and cultural period. In addition, remains of ceremonial activities, such as offerings of pottery vessels, ritual banquets, were also identified [51].

The Lima culture developed funerary patterns characterized either by burials, whereby single or multiple bodies were wrapped with cloth in extended position, or the practice of human sacrifices with no specific position of the bodies, with or without associated pottery and burial offerings [51]. Bioanthropological analysis of human remains at Huaca Pucllana showed a large number of violent deaths among the Lima individuals [51]. Notably, some individuals (i.e. young women), were sacrificed as part of rituals during the last stages of the construction of the Great Pyramid [52]. Huaca Pucllana appears to have been abandoned in the 7^th^ century AD as a result of political and population changes driven by the expansion of the Wari, who arrived from the highland region of Ayacucho [8].

The Wari culture represents the second settlement stratum at Huaca Pucllana during the MH (800–1000 AD). Wari people partly destroyed and re-structured the former ceremonial Lima center at the Great Pyramid, and used the place as an imperial elite cemetery [45]. However, most of the Wari burials were destroyed later on, which altered the distribution of the elements of each burial, and intact mummies were only found in very few cases [49]. Most of the burials from this stratum are characterized by “fardos”. These funerary bundles preserved corpses before burial and are the main distinctive feature found in the upper platforms of the Great Pyramid (Fig 5).

Other diagnostic signs of elite Wari mortuary practices are the presence of clothing, household goods, ritual items and food [53, 54]. Some tombs and burials only contain a single individual, while others can contain more than four bodies. Some cases include human sacrifices such as young children next to the main body, which is described as a typical Wari mortuary practice for important personalities as recently confirmed at the “Castillo de Huarmey” archaeological site [55].

The major cultural transition from the MH to the LIP (1100–1476 AD) involves the collapse of the Wari Empire [2, 3, 56]. From ~1000 AD onward, the Wari power gradually declined as their political center increasingly lost control over cities and the vast Central Andean territories. During this period of crisis, large urban centers such as Cajamarquilla and Pachacamac in the Central Coast were abandoned [21], only to become occupied by the Ychsma culture (1000–1450 AD) soon after.

However, there is no archaeological evidence for an immediate Ychsma occupation at Huaca Pucllana. Instead, some remaining local people (farmers and fishermen) with no particular cultural affiliation settled near the ceremonial center for about 100 years, later to become the social unit called Ychsma. The Ychsma culture marks the third major period at Huaca Pucllana, during which the site was again used for offerings and burials. The Ychsma culture is associated with a characteristic pottery closely associated to funerary patterns that also used the “fardos” as mortuary elements. Similarly to other settlements/locations in the Central Coast, e.g. Pachacamac [57], several elements suggest a reconfiguration of Huaca Pucllana as religious space that was then continuously used during Ychsma times [46].

### Sample description

A total of 115 samples—mostly teeth—from 52 individuals were collected at the Museo del Sitio Huaca Pucllana (S1 Table). Individuals could be associated to either one of the three successive cultures found in Huaca Pucllana: Lima (n = 35), Wari (n = 47) and Ychsma (n = 33) [46, 58]. We followed standard aDNA sampling guidelines, and collected at least two independent samples per individual for replication and authentication purposes [59, 60].

### Radiocarbon dates from Huaca Pucllana samples

Nine samples excavated at the site were sent for radiocarbon analysis at the University of Oxford Radiocarbon Accelerator Unit, England. The dates were then used to confirm the assignment to cultural periods based on archaeological context for all other Huaca Pucllana samples. Radiocarbon dates were calibrated using the Oxcal computer program (v4.2), applying the `IntCal13' dataset—Northern Hemisphere [61]. Dates estimates were also calibrated with the Southern Hemisphere correction (SHCal13) [62]. For each individual sample analyzed, we observed slight differences between dates calibrated with IntCal13 and SHCal13, although there was a strong overlap. However, these differences between calibration methods did not lead to ambiguities regarding cultural assignment (Table 3).

**Table 3:** Radiocarbon dates from selected samples from Huaca Pucllana.

Sample number	Culture	Relative date	hg	ORAU#	Delta 13 C	uncal BP	IntCal13 cal AD	2-sigma	SH13Cal13 cal AD
**10791**	Lima	100–650 AD	A2	OxA-31118	-11.79	1420±29	584–660 (95.4%)	584–660	603–760 (95.4%)
**10817**	Lima	100–650 AD	C1b	OxA-31120	-13.8	1493±30	534–642 (90.6%)	435–642	549–652 (95.4%)
**10734**	Wari	500–1000 AD	C1b	OxA-31422	-12.65	1156±22	776–968 (95.4%)	776–968	891–988 (95.4%)
**10754**	Wari	500–1000 AD	B2b	OxA-31423	-12.61	955±65	974–1220 (95.4%)	974–1220	1016–1264 (95.4%)
**10709**	Ychsma	1100–1440 AD	C1b	OxA-31424	-9.6	745±23	1244–1288 (92.9%)	1226–1288	1271–1315 (72.9%)
**10805**	Ychsma	100–650 AD	D1	OxA-31462	-13,38	762±23	1223–1280 (95.4%)	1223–1280	1231–1379 (95.3%)
**10810**	Ychsma	100–650 AD	C1d	OxA-31119	-12.18	866±28	1149–1249 (80%)	1048–1249	1164–1272 (95.4%)
**10722**	Ychsma	1100–1440 AD	D1	OxA-31425	-12.49	773±24	1221–1278 (95.4%)	1221–1278	1227–1301 (93.9%)

Radiocarbon dates were calibrated using the OxCal Program v.4.2 from University of Oxford Radiocarbon Accelerator Unit.

### Sample preparation and DNA extraction

Samples were processed in a dedicated aDNA facility at the University of Adelaide’s Australian Centre for Ancient DNA (ACAD), Australia. The laboratory employs standardized protocols and infrastructure for aDNA analysis (positive air pressure, UV irradiation and regular cleaning with oxidating agents, e.g. commercial bleach and Decon^®^ to minimize contamination) [63–65].

Samples were decontaminated upon entry in the aDNA laboratory by exposure to UV light. The surface of the samples was gently wiped with 3% bleach and then physically removed by abrasion using a Dremel^®^ drill. A Mikro-dismembrator ball mill (Sartorius) was used to pulverize the sample and 0.2 g of bone powder were subsequently used in DNA extractions. For the extractions, samples were decalcified by incubation in 4 mL of 0.5 M EDTA (pH 8.0) overnight at 37°C and mixed constantly on a rotor mixer. Next, 70 μL Proteinase K (Invitrogen) was added and the lysis mix was incubated for 2 hours at 55°C under constant rotation. DNA was isolated using silicon dioxide solubilized in a Guanidinium buffer (Qiagen), as described previously [66, 67]. DNA was resuspended in 200μL of TE buffer including 0.05% Tween-20 and stored at -20°C until further use.

### Library preparation and hybridization capture of mtDNA

All DNA extracts were initially screened by sequencing the mitochondrial Hypervariable Region (HVR–I), and by genotyping using two multiplex mitochondrial SNP assays, the GenoCoRe22 [68] and the AmericaPlex26 [69]. We selected one DNA extract for genomic libraries preparation from all individuals for which replications of HVR–I sequences and SNP genotypes were successful. DNA libraries and enzymatic clean-up steps were performed in a dedicated contamination-free room at ACAD. Library preparation with barcoded truncated adapters and hybridization capture of mtDNA are described in [27].

### DNA sequencing and sequence assembly

Pooled DNA libraries were sequenced on one Illumina flow cell lane on an Illumina HiSeq2000 at the Australian Cancer Research Foundation (ACRF) Cancer Genomics Facility, Adelaide, South Australia. Post-sequencing processing was performed using a custom pipeline. Raw Illumina reads were processed and filtered by means of demultiplexing of DNA sequences according to their index sequence, using the Illumina program Casava. Further demultiplexing and trimming of internal barcodes was performed using the program Sabre 1.0 (https://github.com/najoshi/sabre). Residual adapter sequences were trimmed and read pairs were merged using Adapter Removal v1.5 [70] with default parameters. Merged reads were mapped to the reference sequence RSRS (Revised Sapiens Reference Sequences) [30] using the mapping program bwa 0.7.5a-r405 [71], and duplicate reads were removed using FilterUniqueSAMCons.py [72]. Ancient DNA damage patterns were assessed using MapDamage v0.3.6 [73].

### Sequence analysis

Read pileups were visually inspected using the Geneious Pro^®^ Software V.6 (Biomatters Ltd) [74] and variants were called against the Reconstructed Sapiens Reference Sequence RSRS (16,569 bp) [30]. Variant calling was based on a minimum coverage of 3x and an initial automated majority call of 75%, followed by independent verification by eye by two researchers (GV and BL) from the read pile-ups to avoid errors due to DNA damage or to locally re-align regions around homopolymeric stretches.

Haplogroup determination was performed using the online database phylotree (mtDNA tree Build 16 [19 Feb 2014]) [31]. We excluded substitutions at nucleotide positions 16182 and 16183, because they are dependent on the presence of C at the position 16189 [75]. For phylogenetic reconstruction and network analysis, we did not consider positions 309.1C(C), 315.1C AC indels at 523 and 524, deletion 3107 and position 16519 according to [31]. The resulting consensus sequences from Huaca Pucllana individuals were also included in a parallel study surveying the wider pre-Columbian mitochondrial genome diversity across South America [43].

### Population genetic and statistical analyses

Haplotype diversity (*h*), nuclear diversity (*π*), pairwise genetic distances *F*_ST_, neutrality test Tajima’s D [76] and Fu’s FS [77] were calculated using the software Arlequin 3.5 [78]. P-values of genetic distances were corrected for multiple comparisons using the *p*.*adjust* function in R 3.2.3 (The R Project for Statistical Computing 2011, https://www.r-project.org/). Median Joining (MJ) networks [79] were built using the program Network version 4.6 (http://www.fluxus-engineering.com) to evaluate genetic relationships among all individuals and to illustrate the extent of haplotype sharing within and among temporal periods and cultures.

We generated multidimensional scaling (MDS) plots to visualize genetic similarities and dissimilarities as measured by fixation indices (Slatkin’s *F*_ST_) [80] in bi-dimensional space using a customized R script (https://www.r-project.org/). Since the mtDNA genome data for modern-day populations is heavily biased towards phylogenetic and phylogeographic questions that result in an excess of specific haplogroups, we had to restrict our analyses to mtDNA (HVR–I) sequences compiled from published sources to describe the genetic relationships between ancient samples from Huaca Pucllana and other ancient individuals (n = 352) from 16 populations, as well as present-day South Americans (n = 1090) from 28 populations (S2 Table).

We used a Fisher exact test to investigate the variation in sub-haplogroup composition between the three cultural groups. Individuals were assigned to sub-haplogroups A2, B2, B2b, C1b, C1c, C1d, D1, and D4/PreD1/D1. The Fisher extact test was computed using the function *fisher*.*test* in R 3.2.3 (http://www.r-project.org) and 10,000 permutations per comparison.

## Supporting Information

S1 Table List of sample details collected from Huaca Pucllana archaeological site. (DOCX)

S2 Table List of populations used to perform the comparative analysis. (DOCX)
