# Supplementary material for: Ancient DNA Analysis Suggests Negligible Impact of the Wari Empire Expansion in Peru’s Central Coast during the Middle Horizon
Source: PLoS One. 2016 Jun 1;11(6):e0155508. doi: 10.1371/journal.pone.0155508 (PMC4889149; doi:10.1371/journal.pone.0155508)
Supplement: S1 Table — (DOCX) [file pone.0155508.s001.docx]

**S1 Table.** List of sample details collected from Huaca Pucllana archaeological site

| **Sample** | **Sample Number** | **Sample** | **Sample Details** | **Collection Notes** |
| --- | --- | --- | --- | --- |
| **10709** | A15 01/02 Muestra 1a | Tooth | M4,8 | Ychsma culture, adult |
| **10710** | A15 01/02 Muestra 1b | Tooth | M3,7 | Ychsma culture, adult |
| **10711** | A15 01/02 Muestra 1c | Hair |  | Ychsma culture, adult |
| **10712** | A0 cf14 ind-1/98 Muestra 2a | Tooth | M3,6 | Ychsma culture, infant |
| **10713** | A0 cf14 ind-1/98 Muestra 2b | Tooth | M4,6 | Ychsma culture, infant |
| **10714** | A0 cf14 01/98 Muestra 2c | Hair |  | Ychsma culture, infant |
| **10715** | A01 CF16/98 Muestra 3a | tooth | C3,3 | Ychsma culture, adult, female |
| **10716** | A01 CF16/98 Muestra 3b | tooth | M2,7 | Ychsma culture, adult, female |
| **10717** | A0 CF15/01 Muestra 4a | tooth | C4,3 | Ychsma culture, adult |
| **10718** | A0 CF15/01 Muestra 4b | tooth | M1,7 | Ychsma culture, adult |
| **10719** | A15 CF36/01 Muestra 5a | tooth | M4,6 | Ychsma culture, adult |
| **10720** | A15 CF36/01 Muestra 5b | tooth | C4,3 | Ychsma culture, adult |
| **10721** | A15 CF36/01 Muestra 5c | Hair |  | Ychsma culture, adult |
| **10722** | A15 Sin Contexto  Muestra 6a | tooth | M4,8 | Ychsma culture, adult |
| **10723** | A15 Sin Contexto Muestra 6b | tooth | M3,8 | Ychsma culture, adult |
| **10724** | A15 Sin Contexto Muestra 7a | tooth | M3,8 | Ychsma culture, adult |
| **10725** | A15 Sin Contexto Muestra 7b | tooth | M4,8 | Ychsma culture, adult |
| **10726** | A3 CF01/04 Muestra 13a | tooth | C3,3 | Ychsma culture, adult |
| **10727** | A3 CF01/04 Muestra 13b | tooth | M3,8 | Ychsma culture, adult |
| **10728** | A0 08/98 Muestra 49a | tooth | M1,8 | Ychsma culture, adult, male |
| **10729** | A0 08/98 Muestra 49b | tooth | M2,8 | Ychsma culture, adult, male |
| **10730** | A15 02/02 Muestra 50 | bone | left femur | Ychsma culture, adult |
| **10731** | A0 56/97 Muestra 51 | bone | right femur | Ychsma culture, adult |
| **10732** | A01 02/02 Muestra 52 | bone | right humerus | Ychsma culture, adult |
| **10793** | A06 82/96 Muestra 35a | Tooth | C2,3 | Ychsma culture, adult  (20-24yrs), male |
| **10794** | A06 82/96 Muestra 35b | Tooth | M2,8 | Ychsma culture, adult  (20-24yrs), male |
| **10795** | A06 82/96 Muestra 35c | Hair |  | Ychsma culture, adult  (20-24yrs), male |
| **10796** | A06 77/96 Muestra 36a | Tooth | C2,3 | Ychsma culture, adult |
| **10797** | A06 77/96 Muestra 36b | Tooth | C1,3 | Ychsma culture, adult |
| **10800** | A06 79/96 Ind1 Muestra 38a | Tooth | M3,7 | Ychsma culture, adult  (17-20yrs), male |
| **10801** | A06 79/96 Ind1 Muestra 38b | Tooth | M4,6 | Ychsma culture, adult  (17-20yrs), male |
| **10804** | A06 79/01 Ind2 Muestra 40a | Tooth | M3,6 | Ychsma culture, adult  (35-40yrs), male |
| **10805** | A06 79/01 Ind2 Muestra 40b | Tooth | M1,7 | Ychsma culture, adult  (35-40yrs), male |
| **10809** | A06 76/96 Muestra 42a | Tooth | M1,8 | Ychsma culture, adult |
| **10810** | A06 76/96 Muestra 42b | Tooth | M2,7 | Ychsma culture, adult |
| **10733** | A20 08/08 Ind2 Muestra 8a | tooth | M3,6 | Wari culture, adult  (30-35yrs), male |
| **10734** | A20 08/08 Ind2 Muestra 8b | tooth | M4,6 | Wari culture, adult  (30-35yrs), male |
| **10735** | A20 08/08 Ind2 Muestra 8c | Hair |  | Wari culture, adult  (30-35yrs), male |
| **10736** | A20 08/08 Ind1 Muestra 9a | tooth | M2,6 | Wari culture, infant  (1-2yrs), female |
| **10737** | A20 08/08 Ind1 Muestra 9b | tooth | M1,6 | Wari culture, infant  (1-2yrs), female |
| **10738** | A20 08/08 Ind3 Muestra 10a | tooth | P1,5 | Wari culture, adult  (40-50yrs), female |
| **10739** | A20 08/08 Ind3 Muestra 10b | tooth | M1,8 | Wari culture, adult  (40-50yrs), female |
| **10740** | A20 08/08 Ind3 Muestra 10c | Hair |  | Wari culture, adult  (40-50yrs), female |
| **10741** | A20 CF003/09 Muestra 11a | tooth | M3,6 | Wari culture, adult  (55-60yrs), female |
| **10742** | A20 CF003/09 Muestra 11b | tooth | M1,7 | Wari culture, adult  (55-60yrs), female |
| **10743** | A20 CF003/09 Muestra 11c | Hair |  | Wari culture, adult  (55-60yrs), female |
| **10744** | A20 11/08 Ind1 Muestra 12a | tooth | M3,7 | Wari culture, adult  (45-55yrs), female |
| **10745** | A20 11/08 Ind1 Muestra 12b | tooth | M4,6 | Wari culture, adult  (45-55yrs), female |
| **10746** | A20 11/08 Ind1 Muestra 12c | Hair |  | Wari culture, adult  (45-55yrs), female |
| **10747** | A20 07/08 Ind6 Muestra 14a | tooth | M8,5 | Wari culture, infant  (6 +/- 2yrs), maybe male |
| **10748** | A20 07/08 Ind6 Muestra 14b | tooth | M6,5 | Wari culture, infant  (6 +/- 2yrs), maybe male |
| **10749** | A20 07/08 Ind4 Muestra 15a | tooth | M2,8 | Wari culture, adult  (35-45yrs), male |
| **10750** | A20 07/08 Ind4 Muestra 15b | tooth | M4,8 | Wari culture, adult  (35-45yrs), male |
| **10751** | A20 07/08 Ind5 Muestra 16a | tooth | M8,5 | Wari culture, infant  (6 +/- 2yrs), male |
| **10752** | A20 07/08 Ind5 Muestra 16b | tooth | M5,5 | Wari culture, infant  (6 +/- 2yrs), male |
| **10753** | A20 05/08 Muestra 17a | tooth | M4,6 | Wari culture, adult  (50-60yrs), male |
| **10754** | A20 05/08 Muestra 17b | tooth | M2,7 | Wari culture, adult  (50-60yrs), male |
| **10755** | A20 05/08 Muestra 17c | Hair |  | Wari culture, adult  (50-60yrs), male |
| **10756** | A20 01/05 Ind5 Muestra 18a | Tooth | C4,3 | Wari culture, adult  (50-60yrs), male |
| **10757** | A20 01/05 Ind5 Muestra 18b | Tooth | M3,8 | Wari culture, adult  (50-60yrs), male |
| **10758** | A20 06/08 Ind1 Muestra 19a | tooth | C2,3 | Wari culture, adult  (16-20yrs), male |
| **10759** | A20 06/08 Ind1 Muestra 19b | tooth | C4,3 | Wari culture, adult  (16-20yrs), male |
| **10760** | A20 06/08 Ind1 Muestra 19c | Hair |  | Wari culture, adult  (16-20yrs), male |
| **10761** | A20 CF8/09 Ind1 Muestra 20a | Tooth | M2,7 | Wari culture, adult  (25-30yrs), male |
| **10762** | A20 CF8/09 Ind1 Muestra 20b | Tooth | M4,7 | Wari culture, adult  (25-30yrs), male |
| **10763** | A20 14/08 Muestra 21a | Tooth | M4,7 | Wari culture, adult  (25-30yrs), female |
| **10764** | A20 14/08 Muestra 21b | Tooth | M3,8 | Wari culture, adult  (25-30yrs), female |
| **10765** | A20 18/08 Muestra 22a | tooth | M3,8 | Wari culture, adult  (45-50yrs), female |
| **10766** | A20 18/08 Muestra 22b | tooth | M4,6 | Wari culture, adult  (45-50yrs), female |
| **10767** | A20 18/08 Muestra 22c | Hair |  | Wari culture, adult  (45-50yrs), female |
| **10768** | A20 17/08 Muestra 23a | Tooth | M2,7 | Wari culture, adult  (35-40yrs), male |
| **10769** | A20 17/08 Muestra 23b | Tooth | M1,7 | Wari culture, adult  (35-40yrs), male |
| **10770** | A20 02/07 Ind2 Muestra 24a | Tooth | M1,6 | Wari culture, adult  (50-60yrs), male |
| **10771** | A20 02/07 Ind2 Muestra 24b | Tooth | C2,3 | Wari culture, adult  (50-60yrs), male |
| **10772** | A20 01/09 Ind1 Muestra 25a | Tooth | M2,7 | Wari culture, adult |
| **10773** | A20 01/09 Ind1 Muestra 25b | Tooth | P2,5 | Wari culture, adult |
| **10774** | A20 01/09 Ind2 Muestra 26a | Tooth | M1,6 | Wari culture, adult, female |
| **10775** | A20 01/09 Ind2 Muestra 26b | Tooth | P1,5 | Wari culture, adult, female |
| **10776** | A20 04/07 Ind1 Muestra 27a | Tooth | M2,6 | Wari culture, adult  (35-45yrs), male |
| **10777** | A20 04/07 Ind1 Muestra 27b | Tooth | M3,8 | Wari culture, adult  (35-45yrs), male |
| **10778** | A20 04/07 Ind2 Muestra 28a | Tooth | M4,6 | Wari culture, subadult  (12-15yrs), maybe female |
| **10779** | A20 04/07 Ind2 Muestra 28b | Tooth | M3,6 | Wari culture, subadult  (12-15yrs), maybe female |
| **10780** | A18 02/09 Muestra 29a | Tooth | M3,7 | Lima culture, adult  (25-30yrs), male |
| **10781** | A18 02/09 Muestra 29b | Tooth | M3,3 | Lima culture, adult  (25-30yrs), male |
| **10782** | A18 02/09 Muestra 29c | Hair |  | Lima culture, adult  (25-30yrs), male |
| **10783** | A06 65 Ind2 Muestra 30a | Tooth | M3,8 | Lima culture, adult  (30-40yrs), female |
| **10784** | A06 65 Ind2 Muestra 30b | Tooth | M1,6 | Lima culture, adult  (30-40yrs), female |
| **10785** | A06 90 Ind2 Muestra 31a | Tooth | M1,6 | Lima culture, adult  (35-40yrs), male |
| **10786** | A06 90 Ind2 Muestra 31b | Tooth | M2,8 | Lima culture, adult  (35-40yrs), male |
| **10787** | A06 90 Ind1 Muestra 32a | Tooth | M1,7 | Lima culture, adult  (40-45yrs), male |
| **10788** | A06 90 Ind1 Muestra 32b | Tooth | M2,6 | Lima culture, adult  (40-45yrs), male |
| **10789** | A20 05/09 Muestra 33a | Tooth | C3,3 | Lima culture, adult  (20-40yrs), male |
| **10790** | A20 05/09 Muestra 33b | Tooth | P4,5 | Lima culture, adult  (20-40yrs), male |
| **10791** | A20 03/07 Ind1 Muestra 34a | Tooth | P4,4 | Lima culture, adult  (30-40yrs), female |
| **10792** | A20 03/07 Ind1 Muestra 34b | Tooth | M4,6 | Lima culture, adult  (30-40yrs), female |
| **10798** | A06 84/01 Ind2 Muestra 37a | Tooth | M3,7 | Lima culture, adult  (30-35yrs), female |
| **10799** | A06 84/01 Ind2 Muestra 37b | Tooth | M2,6 | Lima culture, adult  (30-35yrs), female |
| **10802** | A06 95/96 Muestra 39a | Tooth | M3,7 | Lima culture, adult  (50-55yrs), female |
| **10803** | A06 95/96 Muestra 39b | Tooth | P2,4 | Lima culture, adult  (50-55yrs), female |
| **10806** | A06 102/96 Muestra 41a | Tooth | M3,6 | Lima culture, adult |
| **10807** | A06 102/96 Muestra 41b | Tooth | M4,6 | Lima culture, adult |
| **10808** | A06 102/96 Muestra 41c | Hair |  | Lima culture, adult |
| **10811** | A18 02/02 Muestra 43a | Tooth | M1,7 | Lima culture, adult |
| **10812** | A18 02/02 Muestra 43b | Tooth | M3,7 | Lima culture, adult |
| **10813** | A15 06/00 Muestra 44a | Tooth | M3,7 | Lima culture, adult |
| **10814** | A15 06/00 Muestra 44b | Tooth | M4,7 | Lima culture, adult |
| **10815** | A06 75/01 Ind2 Muestra 45a | Tooth | P2,5 | Lima culture, adult  (35-45yrs), female |
| **10816** | A06 75/01 Ind2 Muestra 45b | Tooth | P1,5 | Lima culture, adult  (35-45yrs), female |
| **10817** | A06 01/02 Muestra 46a | Tooth | M4,7 | Lima culture, adult |
| **10818** | A06 01/02 Muestra 46b | Tooth | M3,7 | Lima culture, adult |
| **10819** | A06 75/01 Ind1 Muestra 47a | Tooth | M1,7 | Lima culture, adult  (20-30yrs), female |
| **10820** | A06 75/01 Ind1 Muestra 47b | Tooth | M2,7 | Lima culture, adult  (20-30yrs), female |
| **10821** | A6 68/96 Muestra 48a | Tooth | M6,5 | Lima culture, adult |
| **10822** | A6 68/96 Muestra 48b | Tooth | M5,5 | Lima culture, adult |
| **10823** | A6 68/96 Muestra 48c | Hair |  | Lima culture, adult |
