# Supplementary material for: Ancient DNA Analysis Suggests Negligible Impact of the Wari Empire Expansion in Peru’s Central Coast during the Middle Horizon
Source: PLoS One. 2016 Jun 1;11(6):e0155508. doi: 10.1371/journal.pone.0155508 (PMC4889149; doi:10.1371/journal.pone.0155508)
Supplement: S2 Table — (DOCX) [file pone.0155508.s002.docx]

**S2 Table.** List of populations used to perform the comparative analysis

| **MODERN POPULATIONS** | **n** | **Country** | **Region** | **Reference** |
| --- | --- | --- | --- | --- |
| Arequipa | **22** | Peru | Central Andean | [[1](#_ENREF_1)] |
| San Martin | **21** | Peru | Central Andean | [[1](#_ENREF_1)] |
| Tayacaja | **59** | Peru | Central Andean | [[1](#_ENREF_1)] |
| Ancash | **33** | Peru | Central Andean | [[2](#_ENREF_2)] |
| Puno_Quechua | **30** | Peru | Central Andean (Highland) | [[3](#_ENREF_3)] |
| Yungay | **36** | Peru | Central Andean | [[3](#_ENREF_3)] |
| Tupe | **16** | Peru | Central Andean | [[3](#_ENREF_3)] |
| Puno_Aymara | **14** | Peru | Central Andean (Highland) | [[3](#_ENREF_3)] |
| Titicaca_Quechua | **37** | Peru | Central Andean (Highland) | [[4](#_ENREF_4)] |
| Titicaca_Aymara | **20** | Peru | Central Andean (Highland) | [[4](#_ENREF_4)] |
| Titicaca_Uros | **7** | Peru | Central Andean (Highland) | [[4](#_ENREF_4)] |
| Gran Chaco | **204** | Paraguay | Gran Chaco | [[5](#_ENREF_5)] |
| Mapuche | **34** | Chile | South Andean | [[6](#_ENREF_6)] |
| Pehuenche | **24** | Chile | South Andean | [[6](#_ENREF_6)] |
| Yaghan | **15** | Chile | Tierra de Fuego | [[6](#_ENREF_6)] |
| Mapuche1 | **18** | Chile | South Andean | [[7](#_ENREF_7)] |
| Yamana | **21** | Chile | Tierra de Fuego | [[7](#_ENREF_7)] |
| Tehuelche | **23** | Chile | South Andean | [[7](#_ENREF_7)] |
| Atacameño | **28** | Chile | Central Andean | [[7](#_ENREF_7)] |
| Aymara | **39** | Chile | Central Andean | [[7](#_ENREF_7)] |
| Pehuenche1 | **41** | Chile | South Andean | [[7](#_ENREF_7)] |
| Huilliche | **47** | Chile | South Andean | [[7](#_ENREF_7)] |
| Quechua_Bol | **93** | Bolivia | Central Andean  (Highland) | [[8](#_ENREF_8)] |
| Aymara_Bol | **97** | Bolivia | Central Andean (Highland) | [[8](#_ENREF_8)] |
| Gaviao | **27** | Brazil | Amazon | [[9](#_ENREF_9)] |
| Xavante | **25** | Brazil | Amazon | [[9](#_ENREF_9)] |
| Zoro | **30** | Brazil | Amazon | [[9](#_ENREF_9)] |
| Arawaken | **29** | Colombia | North Western | [[10](#_ENREF_10)] |
| **ANCIENT POPULATIONS** | **n** | **Country** | **Region** | **Reference** |
| Caverna6_EH | **7** | Peru | Central Andean (Coastal) | [[11](#_ENREF_11)] |
| Palpa_MH | **11** | Peru | Central Andean (Coastal) | [[11](#_ENREF_11)] |
| Palpa_EIP | **56** | Peru | Central Andean (Coastal) | [[11](#_ENREF_11)] |
| Laramate_LIP | **38** | Peru | Central Andean (Highland) | [[11](#_ENREF_11)] |
| Palpa_EH | **26** | Peru | Central Andean (Coastal) | [[11](#_ENREF_11)] |
| MonteGrande_EIP | **11** | Peru | Central Andean (Coastal) | [[11](#_ENREF_11)] |
| Laramate_MH | **39** | Peru | Central Andean (Highland) | [[11](#_ENREF_11)] |
| Palpa_LIP | **11** | Peru | Central Andean (Coastal) | [[11](#_ENREF_11)] |
| MonteGrande_LIP | **11** | Peru | Central Andean (Coastal) | [[11](#_ENREF_11)] |
| Conchopata_MH | **10** | Peru | Central Andean (Highland) | [[12](#_ENREF_12)] |
| Huari_LIP | **17** | Peru | Central Andean (Highland) | [[12](#_ENREF_12)] |
| Pernil Alto | **13** | Peru | Central Andean (Coastal) | [[13](#_ENREF_13)] |
| Tompullo2 | **24** | Peru | Central Andean (Highland) | [[14](#_ENREF_14)] |
| Acchaymarca | **14** | Peru | Central Andean (Highland) | [[15](#_ENREF_15)] |
| Puca | **11** | Peru | Central Andean (Highland) | [[15](#_ENREF_15)] |
| Pampa Grande_EIP-MH" | **19** | Argentina | Central Andean (Highland) | [[16](#_ENREF_16)] |
| Huaca Pucllana Lima | **9** | Peru | Central Andean (Coastal) | This Study |
| Huaca Pucllana  Wari | **10** | Peru | Central Andean (Coastal) | This Study |
| Huaca Pucllana Ychsma | **15** | Peru | Central Andean (Coastal) | This Study |

**References**

1. Fuselli S, Tarazona-Santos E, Dupanloup I. Mitochondrial DNA diversity in South America and the genetic history of Andean highlanders. Mol Biol Evol. 2003;20:1682 - 91.

2. Lewis C, Tito R, Lizarraga B, Stone A. Land, language, and loci: mtDNA in Native Americans and the genetic history of Peru. Am J Phys Anthropol. 2005;127:351 - 60.

3. Lewis C, Lizarraga B, Tito R. Mitochondrial DNA and the peopling of South America. Hum Biol. 2007;79:159 - 78.

4. Barbieri C, Heggarty P, Castri L, Luiselli D, Pettener D. Mitochondrial DNA variability in the Titicaca basin: Matches and mismatches with linguistics and ethnohistory. Am J Hum Biol. 2011;23(1):89-99.

5. Cabana GS, Merriwether DA, Hunley K, Demarchi DA. Is the genetic structure of Gran Chaco populations unique? Interregional perspectives on native South American mitochondrial DNA variation. Am J Phys Anthropol. 2006;131(1):108-19.

6. Moraga M, Rocco P, Miquel J. Mitochondrial DNA polymorphisms in Chilean aboriginal populations: implications for the peopling of the southern cone of the continent. Am J Phys Anthropol. 2000;113:19 - 29.

7. de Saint Pierre M, Bravi CM, Motti JM, Fuku N, Tanaka M, Llop E, et al. An alternative model for the early peopling of southern South America revealed by analyses of three mitochondrial DNA haplogroups. PLoS ONE. 2012;7(9):e43486.

8. Gaya-Vidal M, Moral P, Saenz-Ruales N, Gerbault P, Tonasso L, Villena M, et al. mtDNA and Y-chromosome diversity in Aymaras and Quechuas from Bolivia: different stories and special genetic traits of the Andean Altiplano populations. Am J Phys Anthropol. 2011;145(2):215-30.

9. Ward R, Salzano F, Bonatto S. Mitochondrial DNA polymorphism in three Brazilian Indian tribes. Am J Hum Biol. 1996;8:317 - 23.

10. Melton P, Bricen I. Biological Relationship Between Central and South American Chibchan Speaking Populations: Evidence From mtDNA. Am J Phys Anthropol. 2007;770:753 - 70.

11. Fehren-Schmitz L, Haak W, Machtle B, Masch F, Llamas B, Tomasto Cagigao E, et al. Climate change underlies global demographic, genetic, and cultural transitions in pre-Columbian southern Peru. Proceedings of the National Academy of Sciences. 2014.

12. Kemp BM, Tung TA, Summar ML. Genetic continuity after the collapse of the Wari empire: mitochondrial DNA profiles from Wari and post-Wari populations in the ancient Andes. Am J Phys Anthropol. 2009;140(1):80-91.

13. Fehren-Schmitz L, Reindel M, Cagigao E, Hummel S, Herrmann B. Pre-Columbian population dynamics in coastal southern Peru: A diachronic investigation of mtDNA patterns in the Palpa region by ancient DNA analysis. Am J Phys Anthropol. 2010;141:208 - 21.

14. Baca M, Doan K, Sobczyk M, Stankovic A, Weglenski P. Ancient DNA reveals kinship burial patterns of a pre-Columbian Andean community. BMC Genet. 2012;13(1):30.

15. Baca M, Molak M, Sobczyk M, Weglenski P, Stankovic A. Locals, resettlers, and pilgrims: A genetic portrait of three pre-Columbian Andean populations. Am J Phys Anthropol. 2014.

16. Carnese F, Mendisco F, Keyser C. Paleogenetical study of pre-Columbian samples from Pampa Grande (Salta, Argentina). Am J Phys Anthropol. 2010;141:452 - 62.
